# Specific IgE for foods in a Romanian adult population

**DOI:** 10.1186/2045-7022-1-S1-P30

**Published:** 2011-08-12

**Authors:** Diana Deleanu, Adriana Bujor, Corina Pastor

**Affiliations:** 1 Univ Med & Pharm Iuliu Hatieganu, Allergy, Cluj-Napoca, Romania; 2 Emergency Hospital Octavian Fodor, Allergy, Cluj-Napoca, Romania

## Background

Food allergy is rare diagnosed in Romania. Also the prevalence of allergy is lower in Romanian in comparision with western countries. Food allergy is important but inadequately studied among our population.The aim of our study was to evaluate the presence of specific IgE for foods.

## Material and Methods

Specific IgE for 20 common foods in 432 Romanian adults were evaluatted by MAST in sera. The patients were submited to our allergy department for urticaria (278 pts) and respiratory allergy (rhinits and/or asthma) (154 pts). We evaluated the presence of specific IgE to 20 foods. The foods evaluated were: hazelnuts, peanuts, walnuts, almonds, cow's milk protein (casein and lactoglobulin), egg (white and yolk), celery, carrots, tomatoes, potatoes, cod, crab, orange, apple, wheat four, rye meal, sesame seeds, soya bean.

## Results

In 65 pts (15%) we found at least one specific IgE for foods. In pts with allergic respiratory disease we found a higher precent of positivity for sIgE for foods (43 pts- 28%). In pts with urticaria the presence of sIge to foods was found in 4.88%. The most frequent sIgE were for hazelnuts, carrots, tomato, wheat flour, egg, apple, peanuts, soya bean. There was no correlation between total IgE and the presence of sIgE for foods. The presence of sIgE did not correlate with clinical symptoms of the patients. High level for sIgE to at least one food were present only in 1% of the pts (4 pts).

## Discussion

This is the first evaluation of sIgE to food in Romania in selected patients (either with urticaria either with a knowed respiratory allergy). The presence of sIgE to foods is not equal with food allergy, its presence is needed. The low incidence of sIgE to food in our area might be an explanation for the rare cases of real food allergy.

## Conclusion

The level of sIgE for food is low in our population.

